# Cohort Profile Update: cognition and ageing in the Survey of Health, Ageing and Retirement in Europe Harmonized Cognitive Assessment Protocol (SHARE-HCAP)

**DOI:** 10.1093/ije/dyag184

**Published:** 2026-09-02

**Authors:** Salima Douhou, Michael Bergmann, Yuri Pettinicchi, Marcela C Otero, Arne Bethmann, Giuseppe De Luca, Axel Börsch-Supan

**Affiliations:** Max Planck Institute for Social Law and Social Policy, Munich, Germany; Munich Research Institute for the Economics of Aging and SHARE Analyses, Munich, Germany; SHARE Berlin Institute, Berlin, Germany; HTW Saar University of Applied Sciences, Saarbrücken, Germany; Netherlands Interdisciplinary Demographic Institute-KNAW, The Hague, The Netherlands; University of Groningen, Groningen, The Netherlands; Max Planck Institute for Social Law and Social Policy, Munich, Germany; Munich Research Institute for the Economics of Aging and SHARE Analyses, Munich, Germany; SHARE Berlin Institute, Berlin, Germany; Department of Economics, Business and Statistics (SEAS), University of Palermo, Palermo, Italy; Max Planck Institute for Social Law and Social Policy, Munich, Germany; Munich Research Institute for the Economics of Aging and SHARE Analyses, Munich, Germany; National Bureau of Economic Research, Cambridge, MA, United States

**Keywords:** neuropsychological assessment, ageing populations, cognitive decline, cross-national study

Key Features

- The Survey of Health, Ageing and Retirement in Europe (SHARE) is a cross-national longitudinal survey of people aged 50 years and over that is modelled closely after its sister studies HRS in the USA and ELSA in the UK. It follows respondents every 2 years since 2004.
- SHARE-Harmonized Cognitive Assessment Protocol (HCAP) is a supplemental dataset that provides a large battery of neuropsychological tests that complement the cognitive measures in SHARE. The aim of SHARE-HCAP is to investigate the prevalence and determinants of cognitive impairment and dementia in Europe.
- The tests cover multiple domains of cognition and informant interviews, which are harmonized with other HCAP sister studies around the world, including studies in the USA, UK, India, China, and South Africa.
- SHARE-HCAP respondents are a subsample of the SHARE cohort study and include 2685 individuals aged 65 years and over.
- For collaboration and data access, please check https://www.share-eric.eu/data/data-set-details/share-hcap.

## The original cohort

The Survey of Health, Ageing and Retirement in Europe (SHARE) is a harmonized and multi-national population-based panel study of micro data on health, social, and economic factors [1]. The aim of SHARE is to provide evidence of the rapid ageing process in Europe at individual and population levels. Since 2004, SHARE has conducted interviews every 2 years with more than 140 000 respondents aged 50 years and over plus their partners in 27 European countries and Israel, covering 39 languages. Nine waves of data have been completed and made available to the scientific community. Data collection for the tenth wave has been completed in Fall 2025. Throughout the waves of data collection, countries were added and refreshment samples were drawn to increase the net sample size and compensate for attrition in the longitudinal sample. Across the nine SHARE waves, total response rates ranged from 62% in wave 4 to approximately 30% in wave 8, the latter reflecting the suspension of data collection during the pandemic. Annualized retention rates ranged from 85% to 95%, with more than 100 000 respondents participating in at least two waves, which demonstrates a strong sample stability across waves [2]. Retrospective life histories regarding childhood circumstances, partners, children, accommodation, employment, socio-economic, and health conditions are collected in SHARELIFE. The data were collected in wave 3 (2008–9) and wave 7 (2017) in all 28 countries in a highly structured manner that enhances working with the data in combination with other SHARE datasets. In wave 6 (2015), SHARE collected blood-based biomarkers as additional objective measures of health in 11 European countries and Israel [3, 4].

SHARE is a longitudinal survey infrastructure by and for researchers from various disciplines and works closely with a national team in each of the countries throughout the data generating process. It allows for cross-national and cross-cultural comparative research as every step in the survey life cycle, from sampling to data processing, is ex-ante harmonized across the countries. Additionally, it is harmonized with the US Health and Retirement Study (HRS) [5] and its International Family of Studies, which is a growing network of longitudinal ageing studies around the world.

SHARE data are available free of charge to the scientific community at www.share-eric.eu and are used by researchers and policymakers throughout the world.

## What is the reason for the new data collection?

The rising dementia prevalence is a critical health and economic concern, particularly in rapidly ageing European populations. Although dementia has no cure, prevention through the identification of risk factors is increasingly emphasized [6, 7]. The societal burden is substantial, with high care and significant impacts on families and the social welfare systems [8]. SHARE-Harmonized Cognitive Assessment Protocol (HCAP) was launched to have more detailed and harmonized assessments of cognitive impairment in Europe.

SHARE-HCAP enhances the SHARE study with in-depth assessments modelled after the HCAP, a neuropsychological battery initiated by the HRS [9] and implemented by studies across the globe such as the English Longitudinal Study of Ageing (ELSA) [10, 11], Mexican Health and Aging Study (MHAS) [12, 13], Health and Aging in Africa (HAALSI) [14, 15], and China Health and Retirement Longitudinal Study (CHARLS) [16]. Researchers can better identify cognitive impairment through SHARE-HCAP while exploring health and socio-economic influences on cognitive ageing. Its harmonization with other HCAP studies facilitates cross-national and longitudinal comparisons, offering insights into how policy contexts and life conditions shape cognitive ageing across Europe.

## What will be the new areas of research?

The SHARE-HCAP study fills a significant gap as no other harmonized, population-based data on cognitive functioning exists across Europe, making it invaluable for comparative analysis within Europe and across other ageing studies around the globe.

Contributing to a growing body of research on the classification of cognitive functioning, SHARE-HCAP provides a framework to determine the prevalence of cognitive impairment comparatively within Europe and across other HCAP studies [17–20]. The classification approach is a powerful instrument to capture cognitive functioning across large groups of people within a cross-national, longitudinal framework and helps identify risk factors across diverse populations.

The harmonized design of SHARE-HCAP allows researchers to examine how systemic factors such as healthcare, education, and social support interact with modifiable risk and protective factors to shape cognitive ageing. The inclusion of blood-based biomarkers and life course data enhances the capacity to explore mechanisms underlying cognitive decline.

Furthermore, its diverse population opens new avenues to evaluate the cross-cultural validity of cognitive assessment [21].

## Who is in the cohort?

Five countries have been selected to represent a variety of regions and cultures in Europe: Czechia, Denmark, France, Germany, and Italy. To determine the eligibility for participation in the SHARE-HCAP study, a multi-step procedure was developed where the goal was to have sufficient representation across the cognition continuum.

**Figure 1:**
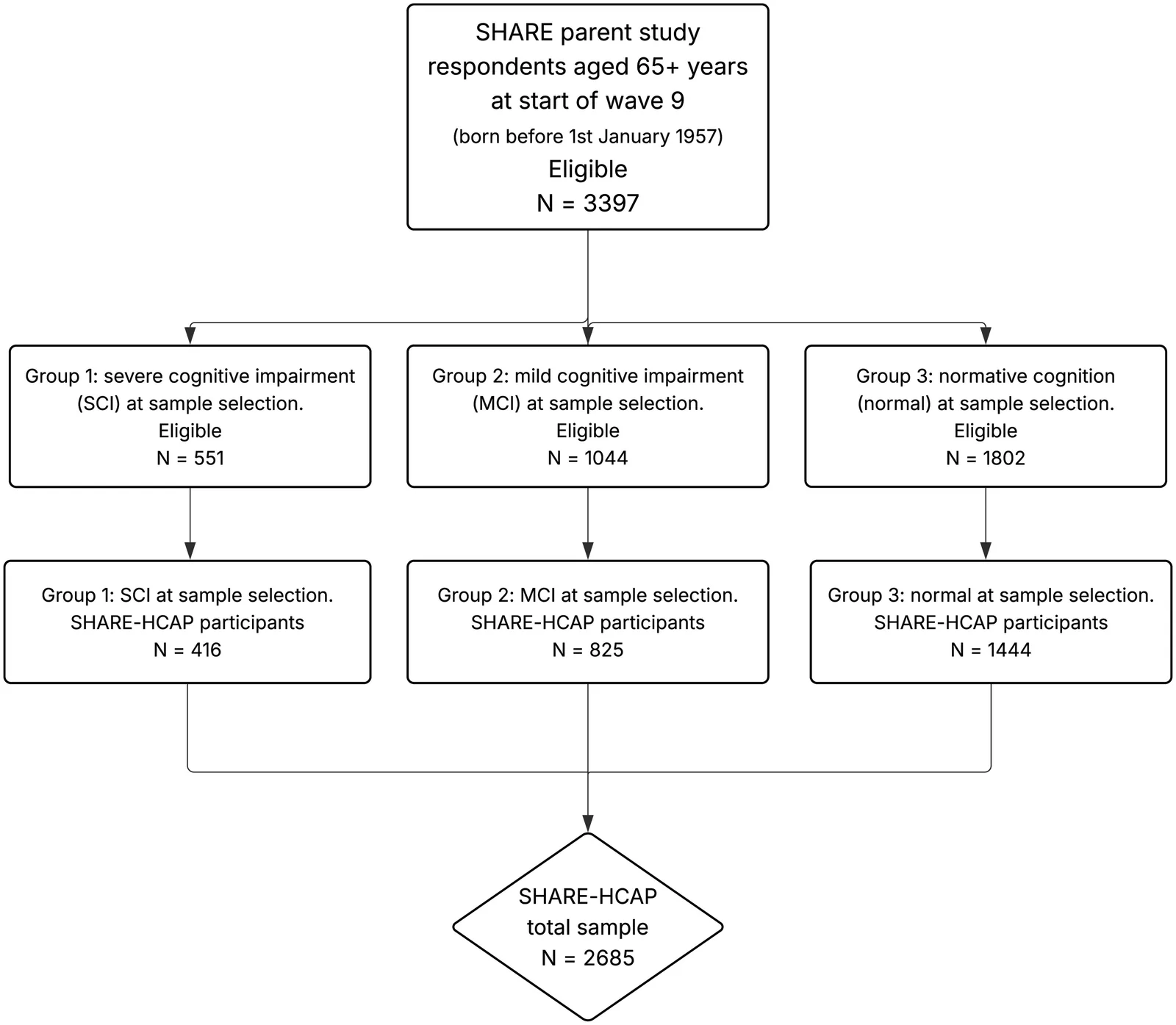
Flowchart of participation in the Survey of Health, Ageing and Retirement in Europe Harmonized Cognitive Assessment Protocol (SHARE-HCAP) study.

In the first step, potential respondents were selected from the SHARE parent study during Wave 9 (2021–22) if they were aged 65 years and over at the start of fieldwork (i.e. born before 1 January 1957) and had completed at least one SHARE interview in the three previous waves to ensure that there was recent baseline information available (Fig. 1). Individuals residing in care or nursing homes were eligible, provided they had the capacity to give informed consent.

In the second step, a selection of respondents was made who were hypothesized to be at greater risk of cognitive impairment based on prior performance on the word recall test in the SHARE parent study and/or a previously reported doctor’s diagnosis of Alzheimer’s disease or related dementia (ADRD) from any of the waves. Like the approach taken in ELSA-HCAP, respondents were categorized into three groups. To account for cross-country variation in word recall performance, country-specific percentiles were used to classify respondents: (1) severe cognitive impairment (SCI, score below the 10th percentile and/or a reported ADRD diagnosis); (2) mild cognitive impairment (MCI, score is between the 10th and 25th percentiles and no reported ADRD diagnosis, and (3) normative cognition (score is above the 25th percentile and no reported ADRD diagnosis.

Third step was to oversample respondents who had been identified as at higher risk of MCI or SCI. Respondents in groups (1) and (2) were fully included, while only a small share of group (3) was fielded at the start of fieldwork to meet country-specific sample size targets. Further, in households with more than one eligible respondent, the individual at higher risk of cognitive impairment was selected to participate. If both were equally at risk, one was randomly selected.

Respondents were also invited to nominate up to three people who were close to them (e.g. partner, family) who would be able to provide information on the respondent’s cognitive functioning and everyday activities; this was the so-called family and friend (F&F) interview.

### Fieldwork and response rates

The fieldwork for SHARE-HCAP [22] took place between May and November 2022, which overlapped with the data collection period of wave 9 [23] of SHARE (October 2021–August 2022). A total of 2685 individuals participated in the study, with 500 or more respondents per country (see Table 1). Most respondents were either in the MCI or normative cognition group at selection. Italy stands out as having the most respondents in the MCI group.

**Table 1:** Response rates and marginal effects in the SHARE-HCAP study, per country and cognition group at selection (eligible and completed interviews).

Cognition group per country, response rates, and average marginal effects								
	1: Severe cognitive impairment			2: Mild cognitive impairment			3: Normative cognition	Overall sample
Country	Participants/eligible (%)	AME^a^ (95% CI)	*P*	Participants/eligible (%)	AME^a^ (95% CI)	*P*	Participants/eligible (%)	Participants/eligible (%)
Czechia	84/136 (61.8)	−10.02 (−19.57, −0.48)	.04	188/259 (72.6)	0.80 (−6.54, 8.14)	.83	229/319 (71.8)	501/714 (70.2)
Denmark	62/85 (72.9)	−6.49 (−16.54, 3.56)	.21	90/112 (80.4)	0.92 (−7.20, 9.05)	.82	421/530 (79.4)	573/727 (78.8)
France	63/80 (78.8)	−3.43 (−13.14, 6.28)	.49	133/176 (75.6)	−6.61 (−13.97, 0.75)	.08	332/404 (82.2)	528/660 (80.0)
Germany	97/121 (80.2)	−1.81 (−9.84, 6.22)	.66	118/158 (74.7)	−7.29 (−15.04, 0.45)	.06	332/405 (82.0)	547/684 (80.0)
Italy	110/129 (85.3)	−5.01 (−12.80, 2.79)	.21	296/339 (87.3)	−2.96 (−8.96, 3.03)	.33	130/144 (90.3)	536/612 (87.6)
Total	416/551 (75.5)	−4.63 (−8.67, −0.60)	.02	825/1044 (79.0)	−1.11 (−4.19, 1.97)	.48	1444/1802 (80.1)	2685/3397 (79.0)

a Measuring change in response probabilities of a cognition group (severe cognitive impairment or mild cognitive impairment) relative to the normative group.

SHARE-HCAP: Survey of Health, Ageing and Retirement in Europe Harmonized Cognitive Assessment Protocol; AME: average marginal effects; CI: confidence interval; *P: P*-value.

The response rate in the overall SHARE-HCAP sample varies between 70.2% in Czechia and 87.6% in Italy (difference: *P *< 0.001, 95% confidence interval [CI]: 13.2, 21.7). Across other countries, pairwise differences are much smaller. Table 1 reports differences in response rates across the participating countries and cognition groups at selection for participation. Overall, respondents who were classified as SCI have about 5% lower response probability (*P *= 0.02, 95% CI: −8.7, −0.6) compared to the normative group, while the difference between MCI and the normative group is negligible (*P *= 0.48, 95% CI: −4.2, 2.0). At the country level, Czechia’s respondents in the SCI group have a 10% lower response probability (*P *= 0.04, 95% CI: −19.6, −0.5) compared to the normative group, whereas no substantial differences were found across the cognition groups in the other SHARE-HCAP countries (*P *> 0.05, 95% CIs include 0). After adjusting for covariates like sex and age, the difference in Czechia drops to 0.3% (*P *= 0.94, 95% CI: −7.6, 8.2) and the remaining estimates become smaller and less precise (Supplementary Table S1, *P *> 0.05, 95% CIs include 0).

Table 2 presents SHARE-HCAP response rates across cognition groups and sociodemographic characteristics. Estimates from probit models show that respondents classified as SCI have a lower response probability, across sex, education, and residence type (*P *< 0.05, 95% CIs not including 0). Response probabilities for the MCI group were comparable to the normative group (*P *> 0.05, 95% CIs include 0). After adjusting for covariates, the differences in response probability across cognition groups were attenuated (Supplementary Table S2). See Supplementary Table S3 for a comparison of the sociodemographic characteristics of the SHARE-HCAP sample with the same country subsample from wave 9.

**Table 2:** Response rates in the SHARE-HCAP study across demographic characteristics and cognition groups at selection (eligible and completed interviews).

		Demographic characteristics and cognition groups, response rates						
		1: Severe cognitive impairment			2: Mild cognitive impairment			3: Normative cognition
Demographic characteristics		Participants/eligible (%)	AME^a^ (95% CI)	*P*	Participants/eligible (%)	AME^a^ (95% CI)	*P*	Participants/eligible (%)
Sex	Female	220/304 (72.4)	−7.23 (−12.82, −1.63)	.01	419/543 (77.2)	−2.43 (−6.73, 1.87)	.27	823/1034 (79.6)
	Male	196/247 (79.4)	−1.51 (−7.27, 4.26)	.61	406/501 (81.0)	0.18 (−4.24, 4.60)	.94	621/768 (80.9)
Age	65–74	82/111 (73.9)	−7.23 (−15.74, 1.28)	.10	289/363 (79.6)	−1.49 (−6.26, 3.28)	.54	854/1053 (81.1)
	75–84	190/242 (78.5)	0.08 (−6.09, 6.25)	.98	391/483 (81.0)	2.52 (−2.34, 7.37)	.31	451/575 (78.4)
	85+	144/198 (72.7)	−7.16 (−15.76, 1.44)	.10	145/198 (73.2)	−6.65 (−15.23, 1.92)	.13	139/174 (79.9)
Education	Low	217/296 (73.3)	−7.68 (−14.00, −1.35)	.02	436/546 (79.9)	−1.13 (−6.23, 3.96)	.66	328/405 (81.0)
	Medium	148/187 (79.1)	−0.67 (−7.16, 5.83)	.84	268/343 (78.1)	−1.68 (−6.91, 3.56)	.53	597/748 (79.8)
	High	50/67 (74.6)	−5.22 (−16.09, 5.65)	.35	121/153 (79.1)	−0.76 (−7.91, 6.39)	.83	515/645 (79.8)
Residence	Community	396/526 (75.3)	−4.77 (−8.90, −0.64)	.02	809/1020 (79.3)	−0.74 (−3.84, 2.36)	.64	1425/1780 (80.1)
	Nursing home	20/25 (80.0)	−6.36 (−27.61, 14.88)	.56	16/24 (66.7)	−19.70 (−43.39, 4.00)	.10	19/22 (86.4)
Wave 9 interview	Self	391/478 (81.8)	−0.44 (−4.34, 3.46)	.82	800/970 (82.5)	0.23 (−2.76, 3.22)	.88	1431/1740 (82.2)
	Proxy^b^	23/35 (65.7)	NA	NA	16/28 (57.1)	NA	NA	3/3 (100.0)
ADL difficulties	None	284/349 (81.4)	−0.90 (−5.41, 3.60)	.69	664/802 (82.8)	0.52 (−2.72, 3.75)	.75	1272/1546 (82.3)
	≥1	128/162 (79.0)	−3.55 (−11.78, 4.68)	.40	150/194 (77.3)	−5.24 (−13.19, 2.70)	.20	161/195 (82.6)
IADL difficulties	None	206/247 (83.4)	0.51 (−4.51, 5.53)	.84	566/685 (82.6)	−0.26 (−3.69, 3.17)	.88	1216/1467 (82.9)
	≥1	206/264 (78.0)	−1.17 (−8.10, 5.76)	.74	248/311 (79.7)	0.55 (−6.02, 7.11)	.87	217/274 (79.2)

a Measuring change in response probabilities of a cognition group (severe cognitive impairment [SCI], or mild cognitive impairment [MCI]) relative to the normative group.

b Interaction between proxy interview and SCI and MCI (relative to normative group) could not be estimated because of sparse data.

SHARE-HCAP: Survey of Health, Ageing and Retirement in Europe Harmonized Cognitive Assessment Protocol; AME: average marginal effects; CI: confidence interval; *P: P*-value; NA: not available; ADL: activities of daily living; IADL: instrumental activities of daily living.

A total of 2281 individuals have participated in the F&F interviews, which is an overall response rate of 85.0% (see Table 3). Of these interviews, 407 interviews were conducted in Czechia, 459 in Denmark, 409 in France, 469 in Germany, and 537 in Italy. In 45 cases, only an F&F interview was completed as respondents could not participate due to cognitive health reasons. Response rates range between 75.8% in France and 96.6% in Italy when excluding unmatched F&F interviews (*P *< 0.001, 95% CI: −24.8, −16.9).

**Table 3:** Demographic characteristics and response rates of SHARE-HCAP Family and Friend interviews.

		F&F interviews *N* = 2281	
Demographic characteristics		*n*	%
Sex	Female	1532	67.3
	Male	744	32.7
Age	<55	467	20.6
	55–64	451	19.9
	65–74	749	33.0
	75–84	507	22.3
	85+	95	4.2
Interview mode	Face-to-face	1296	57.1
	Telephone	973	42.9
Relation to respondent	Spouse/partner	1230	54.1
	Child	613	26.9
	Grandchild	22	1.0
	Sibling	69	3.0
	Parent	17	0.7
	Friend	171	7.5
	Guardian	1	0.0
	Neighbour	42	1.8
	Other	110	4.8
Matched F&F interviews^a^	Czechia	405	80.8
	Denmark	456	79.6
	France	400	75.8
	Germany	457	83.5
	Italy	518	96.6
F&F-only interviews	Czechia	2	
	Denmark	3	
	France	9	
	Germany	12	
	Italy	19	

a These are the interviews that can be matched with a respondent interview. Response rates are calculated as the number of F&F interviews divided over the total number of respondent interviews for a specific country.

SHARE-HCAP: Survey of Health, Ageing and Retirement in Europe Harmonized Cognitive Assessment Protocol; F&F: family and friend.

About two-thirds of F&F interviewees were female, and the majority of interviewees were 65 years or over. Spouses, partners, or children most often completed F&F interviews, likely due to their proximity during the respondent’s face-to-face interview.

### Weights

The calibrated cross-sectional weights for SHARE-HCAP were derived using a two-stage procedure that modelled country-specific response processes. This procedure accounted for design weights, prior participation patterns in SHARE, and SHARE-HCAP stratification variables (i.e. cognition status at selection and one individual per household). The resulting calibrated weights were constructed to satisfy known population margins [24].

## What has been measured?

### Respondent interview

Table 4 presents the SHARE-HCAP respondent battery in order of administration. This multidomain neuropsychological test battery, adapted for the European context, enables cross-study calibration with HRS and its sister studies. The adaptation process for the five countries included consultations with the SHARE-HCAP Project Advisory Board of experts, local cognition experts, and native speakers to ensure cultural relevance.

**Table 4:** SHARE-HCAP respondent and Family and Friend battery.

Domain	SHARE-HCAP respondent tests	SHARE parent study	HCAP sister studies
Orientation, memory (immediate and delayed), visuospatial skills, attention/speed, language fluency	Mini-Mental State Examination (MMSE)	X	✓
Memory (immediate), orientation, language fluency	HRS-Telephone Interview for Cognitive Status (TICS; 3 items: Object naming; naming president)	X	✓
Memory (immediate, delayed and recognition)	CERAD Word List—Recall: Immediate and delayed, Recognition	X	✓
Language fluency	Semantic Fluency (Animal Naming)	✓	✓
Attention/speed, visuospatial	Symbol cancellation test	X	✓
Attention/speed	Timed Backward Counting Task	✓	✓
Orientation, executive functioning, language fluency	Brief Community Screening Instrument for Dementia (CSI-D; 4 items)	X	✓
Memory (immediate, delayed and recognition)	Logical memory (story recall)—immediate, delayed, and recognition	X	✓
Memory (immediate and delayed), visuospatial skills	CERAD Constructional Praxis—immediate and delayed	X	✓
Executive functioning, attention/speed	Symbol Digit Modalities Test (SDMT)	X	✓
Executive functioning	HRS Number Series	X	✓
Executive functioning	Raven’s Standard Progressive Matrices	X	✓
Executive functioning, attention/speed	Trail Making Test (Part A and Part B)	X	✓

	**SHARE-HCAP F&F interview**	**SHARE parent study**	**HCAP sister studies**

Informant report	Jorm Informant Questionnaire on Cognitive Decline in the Elderly (IQCODE)	X	✓
Informant report	Blessed Dementia Rating Scale	X	✓
Informant report	HRS Activities	X	✓
Informant report	10/66 Dementia Research Group Informant Questionnaire (four items)	X	✓
Informant report	CSI-D Cognitive Activities Questionnaire	X	✓

X: cognitive test not included in the study. ✓: cognitive test included in the study.

SHARE-HCAP: Survey of Health, Ageing and Retirement in Europe Harmonized Cognitive Assessment Protocol; F&F: family and friend; HRS: Health and Retirement Study; CERAD: Consortium to Establish a Registry for Alzheimer’s Disease.

Many of the tests were new to SHARE: Mini-Mental State Examination (MMSE) [25, 26], HRS Telephone Interview for Cognitive Status (TICS) [27], Consortium to Establish a Registry for Alzheimer’s Disease (CERAD: word recall and constructional praxis) [27, 28] Symbol Cancellation [29], Brief Community Screening Instrument for Dementia (CSI-D) [30], Logical memory (East Boston Memory test [31] and Wechsler Memory Scale Logical Memory, WMS-IV) [31, 32], symbol digit modalities test (SDMT) [33], HRS Number Series [34], Raven’s Standard Progressive Matrices Test (Raven’s) [35], and Trail Making Test (TMT) [36]. Given the influence of health on cognitive performance, the protocol includes ADL, IADL, self-reported health conditions, and depressive symptoms (EURO-D scale).

### F&F interview

SHARE-HCAP included an F&F interview with someone else who was familiar with the respondent (Table 4) to complement cognitive assessments. The protocol captures changes in general and cognitive health and changes in functional abilities through measures such as ADLs, IADLs, and health conditions, which overlap with measures in the respondent interview and the SHARE parent study. New measures included are: Informant Questionnaire on Cognitive Decline in the Elderly (IQCODE) [37], Blessed Dementia Rating Scale [38], HRS activities, the 10/66 Dementia Research Group Informant Questionnaire [39], and CSI-D Cognitive Activities Questionnaire [39, 40].

## What has it found? Key findings and publications

Between 2020 and 2024, over 200 peer-reviewed publications have used SHARE cognition data (see https://share-eric.eu/publications). SHARE-HCAP studies have looked at dementia prevalence, education and cognitive ageing, and the psychometric properties of the SHARE-HCAP battery [41–43].

A key contribution of SHARE-HCAP is to validate cognition measures in the SHARE parent study, enabling enhanced estimates of MCI and dementia prevalence. In the most harmonized and comprehensive cross-national assessments of cognition in Europe to date, Börsch-Supan *et al*. [41] find higher dementia prevalence rates in the Mediterranean and Southeastern European countries and a much larger variation of cognitive impairment across Europe and Israel than previously known. Dementia prevalence ranges from 4.5% in Switzerland to 22.7% in Spain, and MCI prevalence from 17.2% in Sweden to 31.1% in Portugal. Most of this variation is explained by early-life education and other risk factors such as age and comorbidities.

SHARE-HCAP also advances methodological research on cross-national harmonization of cognitive assessments, enabling integration with other HCAP studies worldwide [44–47], which includes a publication that outlines recommended best practices from HCAP studies for harmonizable cognitive data collection [48].

SHARE-HCAP offers a resource to study risk and protective factors. As an example, Liao *et al*. [49] use data from SHARE-HCAP and CHARLS-HCAP and reveal culturally distinct associations between sleep patterns and cognitive performance in older adults across Europe and China.

## What are the main strengths and weaknesses?

A key strength of SHARE-HCAP is the ex-ante harmonization of in-depth cognitive measures within a cross-national setting, which is closely aligned with HCAP sister studies. In addition, SHARE-HCAP is harmonized with its parent study, which enables researchers to link rich longitudinal data on socioeconomic status, health, and life history with in-depth cognitive assessments. This integration allows for both cross-national comparability and longitudinal tracking of themes around cognitive ageing across a diverse European landscape. SHARE-HCAP offers an unparalleled resource for studying dementia prevalence and cognitive trajectories, making it a cornerstone for evidence-based policy and ageing research in Europe. These data are supplemented with blood-based biomarker data collected in SHARE, which allows linkage to indicators such as cholesterol levels and inflammatory markers [4].

One limitation of the SHARE-HCAP sample is the coverage of only five countries in Europe. As shown in Börsch-Supan *et al*. [41], the linkage with the SHARE parent study makes it possible to validate its cognition measures to have a refined measure of cognition for all European countries.

Another challenge is the limited sample size per country, which can affect statistical power to conduct stratified analyses. However, there is sufficient variability in SHARE-HCAP across the much-used demographic characteristics of age, sex, education, and functional abilities, as shown in the analyses of this paper.

The ambition is to follow-up respondents of SHARE-HCAP to obtain trajectories of cognitive ageing, which will strengthen the evidence base for research on cognitive decline, dementia, and their associated risk factors.

## Can I get hold of the data? Where can I find out more?

SHARE-HCAP and SHARE data are publicly available through the SHARE Research Data Center to registered users for scientific use (https://share-eric.eu/data/data-access) at http://dx.doi.org/10.6103/SHARE.HCAP1.100. Contact Salima Douhou (hcap@mea-share.eu) for the SHARE-HCAP study.

## Ethics approval

Written informed consent was obtained from all individuals and the SHARE and SHARE-HCAP protocols were approved by the Ethics Committee of the Max Planck Institute in Germany. These studies have been performed in accordance with the Declaration of Helsinki.

## Supplementary Material

dyag184_Supplementary_Data

## Data Availability

The data underlying this article are available through the SHARE Research Data Center, at http://dx.doi.org/10.6103/SHARE.HCAP1.100.
