# Supplementary material for: Cohort Profile Update: cognition and ageing in the Survey of Health, Ageing and Retirement in Europe Harmonized Cognitive Assessment Protocol (SHARE-HCAP)
Source: Int J Epidemiol. 2026 Sep 2;55(5):dyag184. doi: 10.1093/ije/dyag184 (PMC13534146; doi:10.1093/ije/dyag184)
Supplement: Supplementary file 1 [file dyag184_supplementary_data.docx]

**Supplementary Material**

Cohort Profile Update: cognition and ageing in The Survey of Health, Ageing and Retirement in Europe Harmonized Cognitive Assessment Protocol (SHARE-HCAP)

Salima Douhou*^1,2^, Michael Bergmann^3,4^, Yuri Pettinicchi^5,6^, Marcela C. Otero^1,2^, Arne Bethmann^3^,

Giuseppe De Luca^7^, Axel Börsch-Supan^1,2,8^

**^1^Max Planck Institute for Social Law and Social Policy, Munich, Germany**
**^2^Munich Research Institute for the Economics of Aging and SHARE Analyses, Munich, Germany**

^3^SHARE Berlin Institute, Berlin, Germany

^4^HTW Saar University of Applied Sciences, Saarbrücken, Germany

^5^Netherlands Interdisciplinary Demographic Institute-KNAW, The Hague, the Netherlands

^6^University of Groningen, Groningen, the Netherlands

^7^University of Palermo, Palermo, Italy **^8^National Bureau of Economic Research, Cambridge, Massachusetts, USA**

*Corresponding author: Max Planck Institute for Social Law and Social Policy, Munich Research Institute for the Economics of Aging and SHARE Analyses, Leopoldstrasse 139, 80804, Munich, Germany.

E-mail: [douhou@mea.mpisoc.mpg.de](mailto:douhou@mea.mpisoc.mpg.de)

**Supplementary tables**

Supplementary Table S1: Predicted response probabilities in the SHARE-HCAP study, per country and cognition group at selection.

Supplementary Table S2: Adjusted response probabilities in the SHARE-HCAP study across demographic characteristics and cognitive status at selection.

Supplementary Table S3: Demographic characteristics of the SHARE parent and SHARE-HCAP sample.

**Supplementary Table S1:** Predicted response probabilities in the SHARE-HCAP study, per country and cognition group at selection.

|  | **Cognition groups, predicted response probabilities** | | | | | | | | | | | | | |  |
| --- | --- | --- | --- | --- | --- | --- | --- | --- | --- | --- | --- | --- | --- | --- | --- |
|  | **1: Severe cognitive impairment (SCI)** | | | | | **2: Mild cognitive impairment (MCI)** | | | | | | **3: Normative** | **Overall sample** | |  |
| **Country** | Predicted^a^ % | AME^b^ | 95% CI | | *P* | Predicted^a^ % | AME^b^ | 95% CI | | *P* | Predicted^a^ % | | | Predicted^a^ % | |
| Czechia | 69.3 | 0.31 | -7.55 | 8.18 | 0.94 | 72.0 | 2.97 | -3.09 | 9.04 | 0.34 | 69.0 | | | 70.2 | |
| Denmark | 74.7 | -4.52 | -15.05 | 6.02 | 0.40 | 80.2 | 0.98 | -7.18 | 9.14 | 0.81 | 79.2 | | | 78.8 | |
| France | 78.6 | -3.61 | -14.47 | 7.26 | 0.52 | 75.2 | -7.02 | -14.98 | 0.94 | 0.08 | 82.2 | | | 79.9 | |
| Germany | 82.4 | 1.74 | -6.85 | 10.34 | 0.69 | 76.1 | -4.55 | -12.32 | 3.22 | 0.25 | 80.7 | | | 79.9 | |
| Italy | 87.6 | -1.33 | -9.57 | 6.92 | 0.75 | 87.1 | -1.84 | -8.51 | 4.83 | 0.59 | 88.9 | | | 87.6 | |
| *Total* | *78.5* | *-0.69* | *-4.75* | *3.37* | *0.74* | *79.1* | *-0.08* | *-3.22* | *3.05* | *0.96* | *79.2* | | | *79.0* | |

SHARE-HCAP: Survey of Health, Ageing and Retirement in Europe Harmonised Cognitive Assessment Protocol. AME: average marginal effects. CI: confidence interval. P: p-value.

^a^ Predicted % are adjusted response probabilities estimated from a probit model, which includes country dummies, cognition group, age, sex, education, residence type, proxy interview, activities of daily living (ADL), instrumental activities of daily living (IADL).

^b^ Average marginal effects, measuring change in response probabilities of a cognition group (SCI or MCI) relative to the normative group.

**Supplementary Table S2**: Adjusted response probabilities in the SHARE-HCAP study across demographic characteristics and cognitive status at selection.

|  |  | **Cognition grouping & demographic characteristics, adjusted response probabilities** | | | | | | | | | | |  |
| --- | --- | --- | --- | --- | --- | --- | --- | --- | --- | --- | --- | --- | --- |
|  |  | **1: SCI** |  |  |  |  | **2: MCI** |  |  |  |  | **3: Normative** | |
| **Demographic**  **characteristics** | | Predicted^a^ % | AME^b^ | 95% CI | | *P* | Predicted^a^ % | AME^b^ | 95% CI | | *P* | Predicted^a^ % | |
| Sex | Female | 77.0 | -1.56 | -5.70 | 2.57 | 0.46 | 76.5 | -2.03 | -5.34 | 1.28 | 0.23 | 78.5 | |
|  | Male | 80.0 | -1.50 | -5.47 | 2.47 | 0.46 | 79.6 | -1.95 | -5.12 | 1.22 | 0.23 | 81.5 | |
| Age | 65-74 | 79.3 | -1.52 | -5.58 | 2.53 | 0.46 | 78.8 | -1.98 | -5.23 | 1.27 | 0.23 | 80.8 | |
|  | 75-84 | 78.8 | -1.52 | -5.52 | 2.49 | 0.46 | 78.4 | -1.97 | -5.17 | 1.23 | 0.23 | 80.4 | |
|  | 85+ | 74.7 | -1.60 | -5.80 | 2.60 | 0.45 | 74.2 | -2.08 | -5.44 | 1.28 | 0.23 | 76.3 | |
| Education | Low | 78.3 | -1.49 | -5.39 | 2.42 | 0.46 | 77.9 | -1.93 | -5.04 | 1.18 | 0.22 | 79.8 | |
|  | Medium | 78.4 | -1.54 | -5.61 | 2.53 | 0.46 | 78.0 | -2.00 | -5.26 | 1.27 | 0.23 | 80.0 | |
|  | High | 78.2 | -1.60 | -5.87 | 2.66 | 0.46 | 77.7 | -2.08 | -5.51 | 1.35 | 0.23 | 79.8 | |
| Residence | Community | 78.4 | -1.53 | -5.58 | 2.52 | 0.46 | 77.9 | -1.99 | -5.23 | 1.25 | 0.23 | 79.9 | |
|  | Nursing home | 77.5 | -1.70 | -6.19 | 2.78 | 0.46 | 77.0 | -2.21 | -5.82 | 1.40 | 0.23 | 79.2 | |
| Wave 9 interview | Self | 78.6 | -1.52 | -5.55 | 2.51 | 0.46 | 78.2 | -1.98 | -5.20 | 1.25 | 0.23 | 80.1 | |
|  | Proxy | 64.1 | -2.17 | -7.81 | 3.48 | 0.45 | 63.4 | -2.80 | -7.31 | 1.71 | 0.22 | 66.2 | |
| ADL difficulties | None | 78.2 | -1.52 | -5.54 | 2.50 | 0.46 | 77.7 | -1.97 | -5.19 | 1.25 | 0.23 | 79.7 | |
|  | ≥1 | 79.2 | -1.62 | -5.88 | 2.64 | 0.46 | 78.7 | -2.10 | -5.52 | 1.31 | 0.23 | 80.9 | |
| IADL difficulties | None | 78.3 | -1.49 | -5.46 | 2.47 | 0.46 | 77.8 | -1.94 | -5.11 | 1.23 | 0.23 | 79.7 | |
|  | ≥1 | 78.6 | -1.66 | -6.00 | 2.69 | 0.45 | 78.1 | -2.15 | -5.63 | 1.33 | 0.23 | 80.3 | |

SHARE-HCAP: Survey of Health, Ageing and Retirement in Europe Harmonised Cognitive Assessment Protocol. AME: average marginal effects. CI: confidence interval. P: p-value. ADL: activities of daily living. IADL: instrumental activities of daily living.

^a^ Predicted percentages are adjusted response probabilities estimated from a probit model, which includes country dummies, cognition group, age, sex, education, residence type, proxy interview, ADLs, IADLs.

^b^ Average marginal effects, measuring change in response probabilities of a cognition group (SCI or MCI) relative to the normative group.

**Supplementary Table S3**: Demographic characteristics of the SHARE parent and SHARE-HCAP sample.

|  |  | **Sample composition, SHARE-HCAP vs SHARE wave 9** | | | | | |
| --- | --- | --- | --- | --- | --- | --- | --- |
|  |  | *SHARE-HCAP N = 2685* | |  |  | *SHARE wave 9 N = 9956* | |
| **Demographic characteristics** |  | *n* | % | t-stat^a^ | *P* | *n* | % |
| Sex | Female | 1462 | 54.5 | -1.02 | 0.31 | 5531 | 55.6 |
|  | Male | 1223 | 45.5 | 1.02 | 0.31 | 4425 | 44.4 |
| Age | 65-74 | 1225 | 45.6 | 4.87 | 0.00 | 5069 | 50.9 |
|  | 75-84 | 1032 | 38.4 | -2.26 | 0.02 | 3591 | 36.1 |
|  | 85+ | 428 | 15.9 | -3.92 | 0.00 | 1296 | 13.0 |
| Education | Low | 977 | 36.5 | -2.15 | 0.03 | 3397 | 34.2 |
|  | Medium | 1017 | 37.9 | 3.57 | 0.00 | 4145 | 41.8 |
|  | High | 686 | 25.6 | -1.70 | 0.09 | 2383 | 24.0 |
| Residence | Community | 2630 | 98.0 | 4.14 | 0.00 | 9852 | 99.0 |
|  | Nursing home | 55 | 2.0 | -4.14 | 0.00 | 104 | 1.0 |
| Wave 9 | Self | 2622 | 98.4 | -1.97 | 0.05 | 8971 | 97.8 |
|  | Proxy | 42 | 1.6 | 1.97 | 0.05 | 201 | 2.2 |
| ADL difficulties | none | 2220 | 83.5 | 4.41 | 0.00 | 7966 | 86.9 |
|  | ≥1 | 439 | 16.5 | -4.41 | 0.00 | 1206 | 13.1 |
| IADL difficulties | none | 1988 | 74.8 | 5.35 | 0.00 | 7301 | 79.6 |
|  | ≥1 | 671 | 25.2 | -5.35 | 0.00 | 1871 | 20.4 |
| Country | Czechia | 501 | 18.7 | 6.43 | 0.00 | 2445 | 24.6 |
|  | Denmark | 573 | 21.3 | -14.41 | 0.00 | 1081 | 10.9 |
|  | France | 528 | 19.7 | -4.21 | 0.00 | 1616 | 16.2 |
|  | Germany | 547 | 20.4 | 2.70 | 0.01 | 2272 | 22.8 |
|  | Italy | 536 | 20.0 | 5.98 | 0.00 | 2542 | 25.5 |

SHARE-HCAP: Survey of Health, Ageing and Retirement in Europe Harmonised Cognitive Assessment Protocol. P: p-value. ADL: activities of daily living. IADL: instrumental activities of daily living.

^a^T-test statistics of differences in response rates between SHARE-HCAP and SHARE wave 9 respondents.
